# How organizational psychological ownership affects corporate green operations - Based on a social exchange theory perspective

**DOI:** 10.3389/fpsyg.2022.985017

**Published:** 2022-08-05

**Authors:** Qingjin Wang, Renbo Shi, Fengying Zhang, Xueling Wang, Yang Gao

**Affiliations:** ^1^Business School, Qingdao University, Qingdao, China; ^2^Art School, Anshan Normal University, Anshan, China

**Keywords:** organizational psychological ownership, corporate green operations, stakeholder pressure, social exchange theory, organizational psychology

## Introduction

With the gradual promotion of China's high-quality economic development and the concept of harmonious society, the problems of slow economic growth, declining corporate profits, and weak technological innovation caused by the traditional high-energy and high-pollution growth model need to be addressed. The demand for green and low-carbon production from stakeholders, such as government and consumers, has become increasingly strong (Sarkis et al., 2010). In this context, the concept of green operation has become a global issue, and countries have started to focus on and improve the content and function of green operation. Green operation has the dual externalities of reducing environmental damage and promoting sustainable development, which not only improves the efficiency of natural resources use at the source, but also helps companies to prevent and control pollution and reduce the negative impact on the environment (Kitsis and Chen, 2021b). At the same time, green operation practices help to gain the favor of stakeholder groups, such as consumers, and thus gain a competitive advantage and achieve superior environmental returns (Lai and Wong, 2012). Therefore, green operations have become an important tool for companies to enhance their competitiveness, promote high-quality economic development, and address environmental issues.

Our review of the research literature on corporate green operations reveals that scholars have mostly explored corporate green operations activities extensively from the institutional and resource perspectives. For example, Peng et al. (2018) verified that environmental regulations promote corporate environmental responsibility. Hafezi and Zolfagharinia (2018) found that environmental regulations set by the government can drive the development of green products by firms, which ultimately enhances environmental performance. In addition, Khan et al. (2019) found that the use of green energy resources can mitigate the negative impacts of corporate social and environmental sustainability. Although corporate green operations activities are well-discussed in institutional and resource perspectives (de Burgos-Jiménez et al., 2013), relatively little of this literature addresses organizational psychology, and few scholars have explored the impact on corporate green operations from the perspective of organizational psychological ownership. The essence of organizational psychological ownership lies in the individual's occupation and control of the organization, i.e., the individual perceives that the organization belongs to the self itself. Therefore, organizational psychological ownership makes employees naturally feel a sense of responsibility and obligation to the organization (Zhang et al., 2021), and are more likely to reciprocate by performing beneficial reciprocal behaviors for the organization (Guo et al., 2022). Based on this, it is reasonable to assume that employees' organizational psychological ownership will have an impact on corporate green operations activities.

In addition, the relationship between organizational psychological ownership, an attitudinal variable with both affective and cognitive components, and corporate green operations is moderated by some contextual factors (Zhang et al., 2021). With the deterioration of ecological environment and global greening, stakeholders' environmental needs and demands have become important factors that drive companies to adopt environmental protection measures (Surroca et al., 2013; Kitsis and Chen, 2021a). Under stakeholder pressure, employees with higher organizational psychological ownership are more likely to respond to and implement the company's green strategy, produce green products that meet a higher environmental management system, and satisfy more stakeholders' needs (Yu and Ramanathan, 2015; Guerci et al., 2016). Therefore, we draw on the perspective of social exchange theory and introduce the contextual factor of stakeholder pressure as a moderating variable to deeply analyze the relationship between the perspective of organizational psychological ownership on corporate green operations in order to enrich the existing knowledge of social exchange theory and provide empirical references for corporate green operations activities.

## Theoretical review and hypotheses development

### Social exchange theory

In 1958, sociologist Homans formally introduced social exchange theory in his article “Social Behavior as Exchange.” Social exchange theory believes that when individuals exchange resources with others, they decide whether to make the exchange by measuring the perceived benefits and costs in the exchange process. The perceived benefits include extrinsic and intrinsic benefits. Extrinsic benefits are mainly goods, services, or money obtained through exchange, also known as material benefits (Kuvaas et al., 2020). The social honor and pleasure brought by the exchange are intrinsic gains, which can be divided into psychological and social gains. Psychological benefits are the psychological pleasure and satisfaction that individuals get from the exchange process (Shore et al., 2006). The positive evaluation, support, and sense of belonging that an individual receives from exchanging resources with others to satisfy the individual's social interaction needs are called social gains. Organizational psychological ownership satisfies employees' sense of place, efficacy, and self-identity, and employees tend to reciprocate by engaging in reciprocal behaviors that benefit the organization (Shore et al., 2006). Therefore, we believe that social exchange theory can well explain the impact of employees' organizational psychological ownership on corporate green operations.

### Organizational psychological ownership and corporate green operations

With the strengthening of environmental regulations and the increasing environmental awareness of consumers, green operations are becoming more and more important to companies. In the past few years, more and more companies have adopted different environmental strategies to implement green management of their products and processes (Lai and Wong, 2012). At the same time, the literature related to green operations is also increasing significantly, but relatively little of this literature deals with organizational psychology, and few scholars have explored the impact on corporate green operations from the perspective of organizational psychological ownership. Organizational psychological ownership refers to the extent to which employees feel ownership of the organization, which reflects the individual's response to the question “to what extent do I feel that the organization is mine” (Van Dyne and Pierce, 2004). Plerce et al. (2001) state that organizational psychological ownership is the attitude that individuals hold toward the organization. Once employees have developed a sense of ownership of the organization, their sense of responsibility and obligation to the organization will increase accordingly, and they will tend to enhance organizational effectiveness by adopting behaviors that are beneficial to the organization. Social exchange theory also believes that exchange in social life exists not only between individuals, but also between individuals and organizations. Organizational psychological ownership satisfies employees' sense of place, efficacy, and self-identity, and employees will reciprocate by performing reciprocal behaviors that benefit the organization (Guo et al., 2022). Based on this, it is reasonable to assume that employees' organizational psychological ownership has an impact on the green operation of the company.

Individual behavior is influenced by emotions and attitudes. Once employees develop a sense of “my organization,” they naturally develop a sense of responsibility and obligation to the organization, and thus act in a way that is beneficial to the organization (Mehmood et al., 2021). Earlier scholars also pointed out that the feeling of “mine” toward the target can trigger positive behavior of individuals to protect the target. According to social exchange theory, when organizations meet employees' basic needs of ownership, efficacy, and identity, employees will reciprocate by adopting positive behaviors that benefit the organization (Andriotis and Paraskevaidis, 2021). For example, employees with higher organizational psychological ownership can help companies reduce waste in production and operations, reduce the cost of energy consumption and waste disposal, and to some extent achieve internal green management in the company. Meanwhile, Plerce et al. (2001) pointed out that organizational psychological ownership means that employees experience higher organizational self-esteem and self-respect, and they perceive themselves as valuable to the organization and thus behave in a way that benefits the organization. Therefore, employees with higher organizational psychological ownership can help companies produce green products that comply with higher environmental management systems, help companies gain more favor from stakeholder groups (especially consumers), and enhance their competitive advantage in the industry. Based on the above analysis, we believe that employees' organizational psychological ownership will promote the green operation activities of the company.

### Moderating effect of stakeholder pressure

Social psychological research on attitudes suggests that the relationship between individual attitudes and behaviors is not a simple one-to-one correspondence, but is mediated by a large number of contextual variables. Bandura (2006) pointed out that individual behavior is influenced by the interaction of individual and environmental factors, i.e., the external environment can directly influence individual attitudes and behavior on the one hand, and play a moderating role in the link between individual factors and behavior due to differences in individual environmental perceptions on the other hand. Plerce et al. (2001) also suggest that the relationship between organizational psychological ownership, an attitudinal variable with both affective and cognitive components, and corporate green operations can be moderated by contextual factors.

Stakeholder concerns about environmental issues have grown significantly in recent years, and companies are facing increasing environmental pressures from different stakeholder groups. Supply chain stakeholders, especially customers and suppliers, may influence a company's decision to adopt environmental practices (Hofer et al., 2012). Stakeholder pressure may prompt companies to give greater consideration to environmental issues and may encourage them to integrate environmental practices into their management strategies and develop strategies, policies, and practices that are consistent with the organization's environmental goals (Yu and Ramanathan, 2015). Van Dyne and Pierce (2004) argue that employees' sense of ownership of the organization directly influences employees' attitudes and behavior. Under stakeholder pressure, employees with higher organizational psychological ownership are more likely to respond to and implement the company's green strategy, reduce waste in production and operations, improve productivity, and help the company achieve internal green management. At the same time, employees with higher organizational psychological ownership are more likely to integrate stakeholder perspectives into the product design and production process, produce green products that comply with higher environmental management systems, and meet the needs of more stakeholders (Ba et al., 2013). Therefore, we suggest that stakeholder pressure has a positive moderating effect between organizational psychological ownership and corporate green operations.

## Discussion

First, we draw on social exchange theory to construct an operational framework to describe how employee organizational psychological ownership affects the green operations of firms. Corporate green operations have been well-discussed in terms of resources and institutions, but relatively little of this literature addresses organizational psychology. Therefore, this paper explores the impact on corporate green operations activities from the perspective of employee organizational psychological ownership, to some extent filling the research gap of combining organizational psychology and green operations and adding a new explanatory logic to the study of green operations drivers.

Second, previous studies have also ignored how stakeholders moderate the relationship between organizational psychological ownership and corporate green operations activities. We introduced stakeholder stress as a moderating variable and found that stakeholder stress has a positive moderating effect between organizational psychological ownership and corporate green operations. In this way, our study extends the understanding of stakeholder stress on the relationship between organizational psychological ownership and corporate green operations and fills a research gap in the integration of stakeholder and organizational psychology.

## Author contributions

QW contributed to the study conception and design. FZ, XW, and YG conducted literature review. The first draft of the manuscript was written by RS. All authors commented on previous versions of the manuscript and contributed to the article and approved the submitted version.

## Funding

This work was supported by the National Social Science Fund Key Project of China (20AZD095).

## Conflict of interest

The authors declare that the research was conducted in the absence of any commercial or financial relationships that could be construed as a potential conflict of interest.

## Publisher's note

All claims expressed in this article are solely those of the authors and do not necessarily represent those of their affiliated organizations, or those of the publisher, the editors and the reviewers. Any product that may be evaluated in this article, or claim that may be made by its manufacturer, is not guaranteed or endorsed by the publisher.

## References

[B1] Andriotis K. Paraskevaidis P. (2021). Negotiated exchanges in the online hospitality market: hoteliers and hotel managers' perceptions of Booking.com. Int. J. Hosp. Manag. 97:103010. 10.1016/j.ijhm.2021.103010

[B2] Ba S. Lisic L. L. Liu Q. Stallaert J. (2013). Stock market reaction to green vehicle innovation. Prod. Oper. Manag. 22, 976–990. 10.1111/j.1937-5956.2012.01387.x

[B3] Bandura A. (2006). Toward a psychology of human agency. Perspect. Psychol. Sci. 1, 164–180. 10.1111/j.1745-6916.2006.00011.x 26151469

[B4] de Burgos-Jiménez J. Vázquez-Brust D. Plaza-Úbeda J. A. Dijkshoorn J. (2013). Environmental protection and financial performance: an empirical analysis in Wales. Int. J. Oper. Prod. Manag. 33, 981–1018. 10.1108/IJOPM-11-2010-0374

[B5] Guerci M. Longoni A. Luzzini D. (2016). Translating stakeholder pressures into environmental performance – the mediating role of green HRM practices. Int. J. Hum. Resour. Manag. 27, 262–289. 10.1080/09585192.2015.1065431

[B6] Guo M. Y. Brown G. Zhang L. H. (2022). My knowledge: the negative impact of territorial feelings on employee's own innovation through knowledge hiding. J. Organ. Behav. 43, 801–817. 10.1002/job.2599

[B7] Hafezi M. Zolfagharinia H. (2018). Green product development and environmental performance: investigating the role of government regulations. Int. J. Prod. Econ. 204, 395–410. 10.1016/j.ijpe.2018.08.012

[B8] Hofer C. Cantor D. E. Dai J. (2012). The competitive determinants of a firm's environmental management activities: evidence from US manufacturing industries. J. Oper. Manag. 30, 69–84. 10.1016/j.jom.2011.06.002

[B9] Khan S. A. R. Jian C. Zhang Y. Golpîra H. Kumar A. Sharif A. (2019). Environmental, social and economic growth indicators spur logistics performance: from the perspective of South Asian Association for Regional Cooperation countries. J. Clean. Prod. 214, 1011–1023. 10.1016/j.jclepro.2018.12.322

[B10] Kitsis A. M. Chen I. J. (2021a). Do stakeholder pressures influence green supply chain Practices? Exploring the mediating role of top management commitment. J. Clean. Prod. 316:128258. 10.1016/j.jclepro.2021.128258

[B11] Kitsis A. M. Chen I. J. (2021b). Does environmental proactivity make a difference? The critical roles of green operations and collaboration in GSCM. Supply Chain Manag. Int. J. 10.1108/SCM-09-2020-0499. [Epub ahead of print].

[B12] Kuvaas B. Shore L. M. Buch R. Dysvik A. (2020). Social and economic exchange relationships and performance contingency: differential effects of variable pay and base pay. Int. J. Hum. Resour. Manag. 31, 408–431. 10.1080/09585192.2017.1350734

[B13] Lai K. Wong C. W. Y. (2012). Green logistics management and performance: some empirical evidence from Chinese manufacturing exporters. Omega Int. J. Manag. Sci. 40, 267–282. 10.1016/j.omega.2011.07.002

[B14] Mehmood S. A. Malik M. A. R. Akhtar M. S. Faraz N. A. Memon M. A. (2021). Organizational justice, psychological ownership and organizational embeddedness: a conservation of resources perspective. Int. J. Manpow. 42, 1420–1439. 10.1108/IJM-06-2020-0296

[B15] Peng B. Tu Y. Wei G. (2018). Can environmental regulations promote corporate environmental responsibility? Evidence from the moderated mediating effect model and an empirical study in China. Sustainability 10:641. 10.3390/su10030641

[B16] Plerce J. L. Kostova T. Dirks K. T. (2001). Toward a theory of psychological ownership in organizations. Acad. Manag. Rev. 26, 298–310. 10.2307/259124

[B17] Sarkis J. Gonzalez-Torre P. Adenso-Diaz B. (2010). Stakeholder pressure and the adoption of environmental practices: the mediating effect of training. J. Oper. Manag. 28, 163–176. 10.1016/j.jom.2009.10.001

[B18] Shore L. M. Tetrick L. E. Lynch P. Barksdale K. (2006). Social and economic exchange: construct development and validation. J. Appl. Soc. Psychol. 36, 837–867. 10.1111/j.0021-9029.2006.00046.x

[B19] Surroca J. Tribó J. A. Zahra S. A. (2013). Stakeholder pressure on MNEs and the transfer of socially irresponsible practices to subsidiaries. Acad. Manag. J. 56, 549–572. 10.5465/amj.2010.0962

[B20] Van Dyne L. Pierce J. L. (2004). Psychological ownership and feelings of possession: three field studies predicting employee attitude and organizational citizenship behavior. J. Organ. Behav. 25, 439–459. 10.1002/job.249

[B21] Yu W. Ramanathan R. (2015). An empirical examination of stakeholder pressures, green operations practices and environmental performance. Int. J. Prod. Res. 53, 6390–6407. 10.1080/00207543.2014.931608

[B22] Zhang Y. Liu G. Zhang L. Xu S. Cheung M. W. L. (2021). Psychological ownership: a meta-analysis and comparison of multiple forms of attachment in the workplace. J. Manage. 47, 745–770. 10.1177/0149206320917195

